# COVID-19 Microangiopathy: Insights into plasma exchange as a therapeutic strategy

**DOI:** 10.1016/j.htct.2025.103963

**Published:** 2025-08-23

**Authors:** Yigit Baykara, Kaan Sevgi, Yamac Akgun

**Affiliations:** a Stanford University Department of Pathology, Stanford, USA; b Kansas City University of Medicine and Biosciences, Kansas City, MO, USA; c University of Miami Miller School of Medicine, Miami, FL, USA

**Keywords:** COVID-19, Plasma exchange, Thrombotic microangiopathy, Coagulopathy

## Abstract

COVID-19-associated thrombotic microangiopathy has emerged as a severe complication that exacerbates morbidity and mortality in critical cases. Thrombotic microangiopathy, characterized by microvascular thrombosis and endothelial injury, includes conditions like thrombotic thrombocytopenic purpura and atypical hemolytic uremic syndrome. This review investigates therapeutic plasma exchange as a potential strategy to mitigate COVID-19-induced thrombotic microangiopathy, examining its role in removing pro-inflammatory cytokines, immune complexes, and pro-thrombotic factors. Additionally, it highlights the synergistic effects when therapeutic plasma exchange is combined with treatments such as complement inhibitors and immunosuppressants. Preliminary evidence, drawn from case reports and early trials, supports the efficacy of therapeutic plasma exchange in improving outcomes for COVID-19-associated thrombotic microangiopathy. However, larger randomized controlled trials are necessary to definitively establish its place in COVID-19 management, particularly for high-risk and transplant patients with underlying immunological vulnerabilities.

## Introduction

COVID-19, caused by the SARS-CoV-2 virus, has demonstrated a wide range of complications, with thrombotic microangiopathy (TMA) being among the most severe [1]. TMA encompasses conditions such as thrombotic thrombocytopenic purpura (TTP) and atypical hemolytic uremic syndrome (aHUS), both of which involve microvascular thrombosis, organ dysfunction, and poor outcomes [2]. COVID-19-induced endothelial injury plays a central role in the development of these conditions [3]. Endothelial cells, when damaged by the virus, become dysfunctional and initiate coagulation pathways, contributing to TMA development. This endothelial dysfunction is also compounded by the ‘cytokine storm’, where excessive inflammatory responses lead to widespread tissue damage and thrombosis [4].

Understanding the pathophysiology of these conditions in the context of COVID-19 is critical for optimizing treatment. COVID-19 has been shown to trigger complement activation, a vital part of innate immunity, which unfortunately also leads to exaggerated inflammatory and coagulation responses [5]. Studies have highlighted that excessive complement activation leads to microthrombus formation in several organs, such as the lungs and kidneys, contributing to multi-organ failure in critically ill patients [6].

Additionally, imbalances in von Willebrand factor (VWF) and ADAMTS13, a metalloprotease that cleaves VWF, are implicated in COVID-19-related microangiopathy [7]. An increase in circulating VWF, coupled with a deficiency of ADAMTS13, leads to unchecked thrombosis and is particularly evident in TTP and aHUS [7,8]. This imbalance exacerbates thrombus formation, especially in microvascular beds, which contributes to organ damage, particularly in the lungs and kidneys [8]. Thus, addressing these pathophysiological changes is crucial for effective management of COVID-19-associated TMA.

Therapeutic plasma exchange (TPE) has emerged as a potential treatment modality for managing COVID-19-associated TMA. TPE works by removing large volumes of plasma containing pro-inflammatory cytokines, immune complexes, and pro-thrombotic factors, thus addressing the hypercoagulable state seen in these patients [9]. Several case reports and small trials have reported promising results with TPE, especially when used in conjunction with other therapies such as complement inhibitors and immunosuppressants [10,11].

## Pathophysiology of microangiopathy in COVID-19

SARS-CoV-2 primarily affects the endothelium, leading to widespread endothelial dysfunction and microvascular thrombosis. The virus directly infects endothelial cells via the angiotensin-converting enzyme 2 receptor, leading to cellular damage, inflammation, and apoptosis [12]. The resulting endothelial injury activates both immune and coagulation systems, creating a pro-thrombotic state and driving the development of TMA [13]. This process is particularly problematic in critically ill patients, where endothelial injury plays a central role in organ dysfunction and multi-organ failure. Key contributors to this pathophysiology include:

• Cytokine storm: The hyperinflammatory state induced by COVID-19, often referred to as a ‘cytokine storm’, triggers widespread endothelial injury. This storm is characterized by the excessive release of pro-inflammatory cytokines such as interleukin-6, tumor necrosis factor-alpha, and interleukin-1 [14]. These cytokines amplify immune responses and lead to direct endothelial cell activation and damage. This hyperinflammatory state contributes to an uncontrolled immune response that results in microvascular damage and thrombus formation.
• Complement dysregulation: Excessive activation of the complement system is a hallmark of severe COVID-19, exacerbating microthrombus formation [15]. Normally, the complement system functions as part of the innate immune defense, but in COVID-19, overactivation leads to endothelial cell injury and the formation of microthrombi. Specifically, complement factors such as C5b-9, a membrane attack complex, have been found to deposit on endothelial surfaces, leading to cell lysis and inflammation [16]. This dysregulation has been strongly implicated in both thrombotic TTP and aHUS, both of which are forms of TMA.
• VWF and ADAMTS13 imbalance: One of the most critical aspects of COVID-19-associated microangiopathy is the imbalance between VWF and ADAMTS13, a metalloprotease responsible for cleaving VWF to prevent excessive clot formation [7]. COVID-19 induces a significant release of VWF from endothelial cells due to the extensive endothelial activation and damage. Simultaneously, levels of ADAMTS13 decrease, possibly due to consumption or direct inhibition by inflammatory mediators [17]. The resultant accumulation of high-molecular-weight VWF multimers leads to unchecked platelet aggregation and microthrombus formation, particularly in the lungs and kidneys. This imbalance is a driving force behind TTP and aHUS, contributing to severe complications such as acute kidney injury and respiratory failure.

Additionally, other coagulation abnormalities in COVID-19, such as elevated d-dimer levels and fibrin degradation products, further support the role of endothelial dysfunction and the pro-thrombotic state in the development of microangiopathies [18]. This constellation of endothelial injury, immune dysregulation, and coagulation imbalance highlights the complexity of managing COVID-19-induced microangiopathies and underscores the need for targeted therapeutic interventions.

## Clinical manifestations of COVID-19-Associated thrombotic microangiopathy

TMA in COVID-19 patients presents with a wide range of clinical manifestations, affecting multiple organs. The endothelial damage, hyperinflammation, and coagulation abnormalities that characterize COVID-19 contribute to the widespread nature of TMA, impacting the lungs, kidneys, and other organ systems [19]. The severity of these manifestations often correlates with poor clinical outcomes, including multiorgan failure and death.

• Lung involvement: One of the most severe clinical manifestations of COVID-19-associated TMA is lung involvement, which can lead to acute respiratory distress syndrome (ARDS) [20]. SARS-CoV-2-induced endothelial injury in the pulmonary vasculature results in microvascular thrombosis, impaired gas exchange, and widespread inflammation [21]. Studies have shown that microthrombi in the pulmonary capillaries contribute significantly to the severe hypoxia seen in ARDS [22]. Autopsy findings in patients who succumbed to COVID-19 have revealed extensive pulmonary microthrombi, reinforcing the link between microangiopathy and respiratory failure [23]. Furthermore, elevated levels of VWF in the lungs exacerbate clot formation, contributing to the development of TMA-related lung complications such as ARDS.
• Renal involvement: The kidneys are also highly susceptible to damage in COVID-19-associated TMA, with acute kidney injury being a frequent and severe complication [24]. Endothelial injury, along with microvascular thrombosis in the renal vasculature, leads to reduced perfusion, ischemia, and tissue damage. In severe cases, COVID-19 patients may develop aHUS, characterized by hemolysis, thrombocytopenia, and kidney failure [25]. The activation of the complement system and its deposition in the renal microvasculature are critical drivers of kidney injury in COVID-19 patients with TMA [26]. Complement-mediated endothelial injury further exacerbates kidney damage, leading to a rapid decline in renal function and, in many cases, the need for renal replacement therapy [26].
• Systemic microvascular dysfunction: Systemic microvascular dysfunction is a hallmark of severe COVID-19-associated TMA and is often indicative of a poor prognosis [27]. As microthrombi form in various organs, multiorgan failure ensues. The heart, liver, gastrointestinal system, and central nervous system are among the organs affected by systemic microthrombi [28]. For instance, myocardial injury in COVID-19 patients has been attributed to microthrombosis within the coronary vasculature, contributing to heart failure and arrhythmias [29]. Hepatic involvement, manifested by elevated liver enzymes and, in severe cases, liver failure, is also observed due to microvascular injury [30]. Neurological symptoms, such as confusion, delirium, and stroke, have been associated with microthrombi and endothelial dysfunction in the cerebral vasculature [31].

As systemic microvascular thrombosis progresses, patients often develop multi-organ failure, a leading cause of death in critically ill COVID-19 patients [32]. The widespread endothelial dysfunction and uncontrolled microthrombosis place immense strain on multiple organ systems, ultimately leading to irreversible damage. Elevated markers of endothelial damage, such as VWF and fibrinogen, are frequently observed in these patients and serve as indicators of severe disease progression [33].

## Mechanism of therapeutic plasma exchange (TPE)

TPE is a blood filtration method used to treat a variety of immune-mediated and inflammatory conditions by removing large volumes of plasma, which contains pathological components such as pro-inflammatory cytokines, immune complexes, and coagulation factors [34]. In the context of COVID-19-associated TMA, TPE has gained attention due to its ability to address the underlying mechanisms driving the severe inflammatory and thrombotic responses observed in patients [10]. By clearing the responsible agents from the bloodstream, TPE helps restore balance to the immune and coagulation systems, thus mitigating the progression of TMA and improving clinical outcomes [35]. The benefits of TPE in COVID-19-associated TMA stem from its ability to target several key pathological processes:

• Reduction in inflammatory mediators: By removing large volumes of plasma, TPE significantly reduces the circulating levels of these cytokines, thereby helping to control the cytokine storm and reduce the risk of multi-organ failure [36]. Studies have shown that the reduction of cytokines through TPE can lead to clinical improvement in critically ill COVID-19 patients, reducing inflammation and its associated complications [37].
• Restoration of coagulation balance: By clearing excess VWF and replenishing ADAMTS13, TPE helps to restore normal clotting function, reducing the risk of thrombus formation in the lungs, kidneys, and other organs. Furthermore, the removal of immune complexes, which can also contribute to microvascular damage, helps to decrease the pro-thrombotic state [38].
• Mitigation of complement activation: TPE is effective in removing circulating complement proteins, including C3a and C5a, which play a crucial role in the inflammatory and thrombotic processes associated with TMA. By reducing complement activation, TPE can mitigate endothelial damage and reduce the likelihood of microvascular thrombosis.

In addition to these mechanisms, TPE also provides benefits by removing other circulating pathological substances, such as autoantibodies, that may contribute to the progression of TMA [39]. The process typically involves exchanging large volumes of plasma, which is then replaced with donor plasma or albumin. This replenishment helps to restore the levels of essential plasma components, including clotting factors and regulatory proteins, further supporting the patient's recovery [40].

## Evidence supporting therapeutic plasma exchange in COVID-19 microangiopathy

There are several case reports, case series, and studies supporting the benefit of TPE in the treatment of COVID-19-associated TMA (Table 1). Elkayam et al. described a patient who developed TMA in the context of severe COVID-19 infection [41]. The patient received TPE in addition to dexamethasone as standard of care and showed clinical improvement including renal function and declining vasopressor requirement as well as increase in platelets and normalization of hemolysis laboratory results. They attributed to this improvement to cytokine removal of inflammatory mediators which is also suggested by Zhang et al. in their case series of three patients with severe COVID-19 patients with ARDS [42]. Ten days after the treatment, all patients showed resolved respiratory symptoms, improvement of lesions on computed tomography scan, two negative nucleic acid tests and normal body temperature for three days. They claimed that TPE had an acute effect on the treatment of cytokine storm and henceforth led to improvement of the symptoms and radiological/laboratory findings. Similarly, Shi et al. presented a patient with COVID-19 infection who developed respiratory failure, shock and diarrhea and failed to respond to standard therapies [43]. The patient received TPE followed by Intravenous immunoglobulin (IVIG) and showed an immediate clinical response to therapy, supporting the hypothesis that TPE might help signs and symptoms of COVID-19 by removing the cytokines from circulation.

**Table 1:** Evidence supporting therapeutic plasma exchange in COVID-19 microangiopathy.

Study/Authors	Patients and Condition	Treatment Provided	Clinical Results
Elkayam et al. [41]	1 patient with severe COVID-19 and TMA	TPE + dexamethasone	Improved renal function, decreased vasopressor requirement, increased platelet counts, normalization of hemolysis parameters
Zhang et al. [42]	3 patients with severe COVID-19 and ARDS	TPE	Resolution of respiratory symptoms, improved CT lesions, negative nucleic acid tests, normalized temperature
Shi et al. [43]	1 patient with COVID-19, respiratory failure, shock, diarrhea	TPE followed by IVIG	Immediate clinical improvement
Arulkumaran et al. [44]	5 patients with severe COVID-19 complicated by ARDS	TPE	Improved oxygenation and reversal of thrombo-inflammatory markers; high VWf:ADAMTS13 ratio observed
Eriko et al. [45]	1 HIV-positive patient with COVID-19-associated TMA	TPE, glucocorticoids, hemodialysis, antihypertensives	Improved renal function and thrombocytopenia
Vrecko et al. [46]	8 patients: 1 with TTP, 7 with aHUS and COVID-19	TTP patient: TPE, steroids, caplacizumab aHUS patients: TPE ± steroids, C5 complement inhibitor, IVIG	Complete hematologic recovery in TTP patient. Rapid hematologic recovery in all aHUS patients; full renal recovery in only one aHUS patient
Cohen et al. [47]	1 patient with COVID-19-associated TTP	TPE, steroids, caplacizumab	Improved abdominal pain, normalization of platelets, hemoglobin, and ADAMTS13
Tehrani et al. [48]	4 patients with COVID-19-associated TTP (2 treated with TPE)	TPE ± Rituximab	Patient 1: Normalization of hemoglobin and platelets Patient 2: No initial response; later response with Rituximab but eventual death due to hemorrhagic stroke

In a study by Arulkumaran et al., five patients who received TPE for severe COVID-19 complicated by ARDS showed improvements in oxygenation and reversal of the thrombo-inflammatory markers [44]. They identified a high VWf Ag:ADAMTS13 ratio in the patients which is comparable to the parameters seen in TMA. Eriko et al. reported a patient with a past medical history of human immunodeficiency virus, who was diagnosed with TMA secondary to COVID-19 [45]. The patient received TPE, glucocorticoids, hemodialysis, and antihypertensives which contributed to improvements in renal function and thrombocytopenia. Vrecko et al. reported the results of their case series of eight patients with COVID-19-associated TMA including one patient with TTP and seven patients with aHUS [46]. The patient with TTP was treated with TPE, steroids and caplacizumab resulting in complete hematologic recovery. Six patients with aHUS were treated with TPE with or without steroids, C5 complement inhibitor and IVIG while the other patient with aHUS was only treated with steroid and C5 complement inhibitor. While all patients with aHUS showed rapid hematologic recovery, only one of them had full renal function recovery.

Cohen et al. presented a case of TTP in the setting of COVID-19 who was treated with TPE, steroids, and caplacizumab. This patient responded to therapy with improvement of the abdominal pain and normalization of platelets, hemoglobin, and ADAMTS13 [47]. Tehrani et al. reported another case series of four COVID-19-associated TTP patients two of whom were treated with TPE [48]. The first patient responded well to the therapy with normalization of hemoglobin and platelet levels. Even though the second patient did not respond to TPE initially, with the addition of rituximab, hemoglobin and platelet levels became normal although she succumbed to death due to hemorrhagic stroke in her frontal cortex.

## Discussion

The emergence of TMA as a complication of severe COVID-19 has brought renewed attention to the complex interplay between endothelial injury, inflammation, coagulation dysregulation, and immune activation. This review consolidates the current understanding of COVID-19-associated TMA and explores TPE as a targeted intervention.

The pathogenesis of COVID-19-associated TMA appears multifactorial. SARS-CoV-2 infects endothelial cells through the angiotensin-converting enzyme 2 receptor, triggering endothelial dysfunction and subsequent activation of coagulation and complement cascades. The cytokine storm further amplifies these processes, while the imbalance between VWF and ADAMTS13 promotes unchecked microthrombus formation. This cascade results in systemic microvascular injury affecting critical organs such as the lungs, kidneys, heart, and brain.

TPE has shown potential in addressing multiple pathological mechanisms underpinning COVID-19-induced TMA. By removing pro-inflammatory cytokines, immune complexes, complement components, and excess VWF, TPE helps attenuate the hyperinflammatory and prothrombotic state. In addition, the replacement plasma replenishes regulatory proteins such as ADAMTS13 and mitigates ongoing vascular injury.

The clinical evidence supporting the use of TPE in COVID-19-associated TMA remains limited but encouraging. Case reports and small case series have documented favorable outcomes, including rapid hematologic improvement, reversal of organ dysfunction, and improved survival in patients with both TTP and aHUS phenotypes. Notably, these outcomes were observed when TPE was employed in combination with immunosuppressants, steroids, and complement inhibitors, highlighting the importance of multimodal therapy.

Nevertheless, the use of TPE in COVID-19 remains controversial due to a lack of large-scale randomized controlled trials. The heterogeneity of TMA presentations, variability in TPE protocols, and differences in co-interventions make it challenging to draw definitive conclusions. Furthermore, TPE is resource-intensive, limiting its widespread application in resource-limited settings. Careful patient selection and early recognition of TMA features are crucial for optimizing outcomes.

## Conclusion

COVID-19-associated TMA represents a severe manifestation of endothelial and immune dysregulation with significant morbidity and mortality. TPE offers a promising strategy to mitigate the cascade of inflammation, complement activation, and thrombosis characteristic of this condition. Early evidence supports its use, especially in cases mimicking TTP or aHUS, and when integrated with other immunomodulatory therapies. However, robust clinical trials are urgently needed to clarify patient selection criteria, treatment timing, and optimal adjunctive therapies. As our understanding of COVID-19-associated microangiopathy evolves, TPE may emerge as a cornerstone of therapy for selected patients, offering a lifeline in an otherwise devastating disease trajectory.

## Author contributions statement

Dr. Yigit Baykara, Dr. Yamac Akgun and Kaan Sevgi contributed to all aspects of this review, including conceptualization, conducting the literature review, and manuscript preparation.

## Conflicts of interest

The authors declare no conflict of interest.
